# Expanding treatment indications in chronic hepatitis B: Should we treat all patients?

**DOI:** 10.1007/s12072-025-10785-8

**Published:** 2025-02-17

**Authors:** Rex Wan-Hin Hui, Lung-Yi Mak, James Fung, Wai-Kay Seto, Man-Fung Yuen

**Affiliations:** 1 Department of Medicine, The University of Hong Kong, Queen Mary Hospital, Pokfulam Road, Hong Kong, China; 2 State Key Laboratory of Liver Research, The University of Hong Kong, Hong Kong, China

**Keywords:** HBV, HBsAg, HBV DNA, Antiviral, NUCs, Treatment, HCC, Cirrhosis, Liver-related mortality, Guidelines

## Abstract

Nucleos(t)ide analogues (NUCs) are first-line agents for chronic hepatitis B (CHB). Current guidelines provide recommendations for NUC initiation, yet the guidelines are complex and restrictive. Accumulating data on hepatitis B virus (HBV) replication and HBV integration suggests that there are no real quiescent disease phases in CHB, and treatment-ineligible patients in current guidelines still have substantial risks of cirrhosis and hepatocellular carcinoma. Expanding CHB treatment indications can effectively reduce the risks of liver-related complications. Furthermore, treatment indication expansion can be cost-effective, and can simplify care pathways to remove treatment barriers. Potential caveats for treatment expansion include risks of non-compliance, long-term side effects from NUCs, and poor patient acceptability. Nonetheless, these caveats are not insurmountable, and the benefits of treatment expansion outweigh the disadvantages. There is consensus among hepatologists in supporting treatment indication expansion, although expert panels have varying recommendations on treatment strategies. A treat-all approach, which involves treating all CHB patients, has also been proposed. A treat-all strategy is straightforward, and should yield the greatest benefits from a population health perspective. However, the feasibility of new treatment strategies, especially the treat-all approach, is influenced by multiple factors including local epidemiology, healthcare resource availability, and socioeconomic factors. A one-size-fits-all approach is not optimal, and treatment expansion strategies that are tailored based on local data should yield the greatest impact toward hepatitis elimination.

## Introduction

Chronic hepatitis B (CHB) is a key cause of cirrhosis, hepatocellular carcinoma (HCC), and liver-related mortality worldwide [1–3]. In 2019, the World Health Organization (WHO) estimated a global hepatitis B virus (HBV) prevalence of 3.8%, with annual HBV-related mortality of 821,000 [1]. Unlike chronic hepatitis C which can be eradicated by a single and finite course of direct-acting antivirals [4], the complete clearance of HBV from humans has remained elusive due to the existence of covalently closed circular DNA (cccDNA) and viral integration into the host genome [5]. Functional cure — defined as sustained hepatitis B surface antigen (HBsAg) seroclearance with unquantifiable HBV DNA at 24 weeks off treatment, is an achievable yet rare endpoint in CHB [6]. Given the barriers in achieving complete or functional cure, prevention of disease progression and complications remain as the key goals of CHB management.

Currently approved therapies for CHB include nucleos(t)ide analogues (NUCs) and pegylated interferon alpha, with the former being the predominant treatment modality used globally [5]. First-line NUCs — including entecavir (ETV), tenofovir disoproxil fumarate (TDF), and tenofovir alafenamide (TAF), are effective in suppressing viral replication with high barriers of resistance. NUCs also improve patient prognosis through inducing fibrosis regression [7–9] and reducing HCC risks [10, 11]. However, it is estimated that only 2.2% of CHB patients worldwide are receiving treatment [1]. While socioeconomic disparities and healthcare inequalities contribute to low treatment rates [12], the complex HBV treatment guidelines also restrict treatment coverage.

The current HBV treatment guidelines from WHO, the American Association for the Study of Liver Diseases (AASLD), the European Association for the Study of the Liver (EASL), and the Asian-Pacific Association for the Study of the Liver (APASL) are summarized in Table 1.[13–16] In general, HBV DNA levels of 2000 IU/ml or 20,000 IU/ml are used as cut-offs for treatment, depending on alanine aminotransferase (ALT) and other clinical parameters. In HBV patients with cirrhosis, treatment is generally recommended regardless of HBV DNA and ALT levels. Furthermore, treatment is recommended for special scenarios, such as in patients with extrahepatic HBV manifestations, hepatitis D virus (HDV) or human immunodeficiency virus coinfection, or with family history of HCC. [13–16]

**Table 1:** Treatment guidelines by international societies

	Non-cirrhotic	Cirrhotic	Special scenarios for treatment
WHO 2024. [13]	HBV DNA > 2000 IU/ml + ALT > ULN^a^	Detectable HBV DNA + any ALT level	Start treatment regardless of HBV DNA if - Significant liver fibrosis - Presence of coinfections (HCV, HDV, HIV) - Comorbidities (diabetes mellitus or metabolic dysfunction-associated steatotic liver disease) - Immunosuppression - Extrahepatic manifestation
AASLD 2018. [14]	HBeAg positive + HBV DNA > 20,000 IU/ml + ALT > 2 × ULN^b^ or HBeAg negative + HBV DNA > 2000 IU/ml + ALT > 2 × ULN^b^	Detectable HBV DNA + any ALT level	Consider treatment even when not fulfilling full treatment indications - Age > 40 - Family history of cirrhosis or HCC - Extrahepatic HBV manifestation - Significant liver fibrosis - Moderate liver necroinflammation
EASL 2017. [15]	HBV DNA > 2000 IU/ml + ALT > ULN^c^ ± at least moderate necroinflammation/ fibrosis	Detectable HBV DNA + any ALT level	Consider treatment even when not fulfilling full treatment indications - HBeAg positive and > 30 years old - Family history of HCC - Extrahepatic HBV manifestations
APASL 2016. [16]	HBeAg positive + HBV DNA > 20,000 IU/ml + ALT > 2 × ULN^c^ or HBeAg negative + HBV DNA > 2000 IU/ml + ALT > 2 × ULN^c^	Detectable HBV DNA + any ALT level	Consider treatment even when not fulfilling full treatment indications - Age > 35 - Family history of cirrhosis or HCC - Significant liver fibrosis - Moderate liver necroinflammation

*ALT* alanine aminotransferase, *HBeAg* hepatitis B e antigen, *HBV* hepatitis B virus, *HBeAg* hepatitis B e antigen, *HCC* hepatocellular carcinoma, *HCV* hepatitis C virus, *HDV* hepatitis D virus, *HIV* human immunodeficiency virus, *ULN* upper limit normal

^a^ULN defined as 30 U/L for males and 19 U/L for females

^b^ULN defined as 35 U/L for males and 25 U/L for females

^c^ULN defined as 40 U/L

With the need to consider multiple factors including HBV DNA, ALT, hepatitis B e antigen (HBeAg), and liver fibrosis severity, the current HBV treatment guidelines may be challenging to implement by non-specialists. Accumulating evidence is supporting the simplification and expansion of HBV treatment indications for clinical, financial, and logistical reasons. Several expert panels are recommending expansion of treatment indications to varying degrees, with some experts recommending treating all CHB patients [17–24]. This article will review the evidence on treatment indication expansion and discuss whether a treat-all approach should be adopted for CHB.

## Clinical benefits of expanding treatment indications

### HBV DNA and integration as risk factors for liver-related complications

HBV DNA is an important predictor of adverse outcomes in CHB. The REVEAL cohort from Taiwan demonstrated a significant positive correlation between HBV DNA and HCC risks, and patients with HBV DNA > 6 log copies/ml had over tenfold higher HCC risks when compared to patients with HBV DNA < 300 copies/ml [25]. A similar biologic gradient between HBV DNA and adverse outcomes has also been demonstrated for cirrhosis, liver cancer mortality, liver-related mortality, and all-cause mortality respectively. [26–28] Overall, HBV DNA is independently associated with liver-related complications, even after controlling for ALT and other clinical parameters [25–27]. HBV integration into the human genome is another key driver of CHB progression, as HBV integration induces mutations in both viral and human genes to promote hepatocarcinogenesis [29, 30]. Potentially hepatocarcinogenic HBV integrations are detectable in all CHB disease phases, irrespective of hepatitis activity, viremia level, and HBsAg positivity. [31]

### Liver-related complications in indeterminate phase patients

**Fig. 1:**
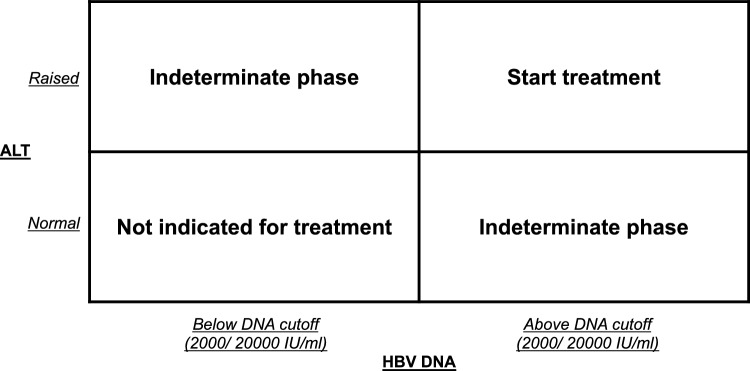
Indeterminate phases in current treatment guidelines

The data on HBV viral replication and HBV integrations suggests that there are no real quiescent disease phases in CHB. While current guidelines attempt to classify patients into distinct disease phases, up to 50% of patients may not be classifiable, and are labeled as indeterminate phase patients (Fig. 1) [32, 33]. NUC therapy is not indicated for indeterminate phase patients in current guidelines, yet these patients have considerable risks of liver-related complications. In a histologic study of 242 indeterminate phase patients from China, 73% had significant liver fibrosis or active inflammation [34]. Comparable results have been replicated in other studies, demonstrating active histologic activity and ongoing liver injury in the indeterminate phase. [35, 36]

In a multicenter study of 3,366 non-cirrhotic CHB patients followed up for 12.5 years, indeterminate phase patients had a 14-fold elevated risk of HCC when compared to patients with inactive disease (defined as normal ALT with HBV DNA < 2000 IU/ml) [37]. Similarly, in a Korean cohort of 6949 non-cirrhotic treatment-naïve patients with ALT < 2 times upper limit normal (ULN), 5.2% developed HCC after 8 years, and a non-linear parabolic risk between HBV DNA and HCC risks was observed [38]. A 2019 multicenter study from Korea further studied HCC occurrence outside treatment guidelines, and the proportion of patients developing HCC outside the APASL, AASLD, and EASL treatment guidelines were 64.0%, 46.0%, and 33.5% respectively. [39]

### Improved clinical outcomes with treatment indication expansion

As NUCs can effectively suppress HBV DNA, expanding treatment indications to include indeterminate phase patients should improve patient outcomes. Through inhibiting intrahepatic HBV DNA replication, NUCs also decrease de novo HBV integrations, and overall integration burden can be reduced with natural hepatocyte turnover [40]. The hypothetical advantages of expanding treatment indications are indeed translatable to improved clinical outcomes. A 2023 multicenter study by Huang et al. studied 855 indeterminate phase patients from 14 centers globally, where 405 patients were on NUCs and 450 were untreated. After balancing clinical characteristics by inverse probability of treatment weighting, the on-treatment patients had significantly lower 10-year and 15-year HCC risks than untreated patients (4% in NUC group vs 15% in untreated group for 10-year risk, 9% in NUC groups vs 19% in untreated group for 15-year risk). The risk disparity remained significant after stratification by age, sex, HBeAg status, HBV DNA, and ALT respectively. NUC therapy remained as an independent protective factor against HCC after controlling for other clinical parameters (Adjusted hazard ratio 0.3, *p* < 0.001). [41]

Apart from HCC reduction, antiviral therapy may confer other clinical benefits in patients outside the current treatment recommendations. The TORCH-B study is a multicenter, double-blind, placebo-controlled, randomized trial that studied CHB patients with HBV DNA > 2000 IU/ml and minimally elevated ALT (between 1–2 times ULN). One hundred sixty patients were randomized to either receive TDF (*n* = 79) or placebo (*n* = 81). After follow-up for 3 years, patients on TDF had significantly lower risks of liver fibrosis progression (26% in TDF group vs 47% in placebo group, relative risk 0.56, *p* = 0.013). The risk of acute HBV flares were also significantly lower in the TDF group (3% in TDF group vs 16% in placebo group, relative risk 0.16, *p* = 0.013) [42]. Concerning the effects of NUCs on HBV integrations, it was shown that patients in the TDF group achieved 3.28-fold reduction in integrations across 3 years, which was significantly higher than the 1.81-fold reduction in placebo patients (*p* = 0037). [43]

Theoretically, treating all CHB patients should minimize the risks of liver-related complications, even in relatively low-risk patients. While the treat-all approach has not been studied in the real-world setting, simulation studies have supported the benefits of treating all patients. Simulation studies from China and the US have both shown a treat-all strategy to be the most effective option for reducing HBV-related complications. In China, it is estimated that a treat-all strategy with 80% coverage up till 2050 can reduce HBV-related HCC and deaths by 79% and 83% respectively. [44] Similarly in the US, a treat-all strategy implemented from 2020 to 2050 is predicted to reduce 169,000 liver-related deaths [45]. The studies on the treat-all strategy are summarized in Table 2.

**Table 2:** Evidence on the treat-all approach

Studies	Study design	Study population	Key findings
Nguyen 2024 [47]	Microsimulation modeling	Gambia – Data from 804 patients in the Prevention of Liver Fibrosis and Cancer in Africa study	• A treat-all approach can avert 9,352 DALYs over the lifetime of 100,000 adults, whereas the EASL 2017 criteria will lead to an excess of 795 DALYs • ICER of the treat-all approach is USD 2149 per DALY, which is not cost-effective in this population (Cost-effectiveness threshold of USD 352)
Razavi-Shearer 2023 [45]	Dynamic Markov modeling	US – Data from HBV infected population from the US	• A treat-all approach can reduce 169,000 liver-related deaths from 2020 to 2050 • The treat-all approach is cost-effective at an annual treatment cost of 2,000 USD, and can be cost-saving at a treatment cost of 750 USD. These are achievable, as the annual treatment cost in the US was as low as 362 USD in 2020
Zhang 2023 [44]	Decision analytic Markov modeling	China – Data from HBV infected population from China	• A treat-all approach with 80% coverage can reduce HBV-related HCC by 79% and HBV-related deaths by 83% up till 2050 • A treat-all approach can avert 1,536,561 QALYs by 2050 at a cost of 7,160 USD by 2050, which is cost-effective in this population
Sanai 2020 [48]	Dynamic Markov modeling	Saudi Arabia – Data from published figures and expert opinion on HBV epidemiology in Saudi Arabia	• A treat-all approach is associated with 50 – 55% reduction in HCC and liver-related deaths. In comparison, attainment of the WHO targets of diagnosing 90% infections and treating 80% of patients by 2030 will only reduce HCC and liver-related deaths by 30 – 35% • The treat-all approach increases the annual healthcare costs by 12 billion USD. This is only cost-effective if treatment costs can be reduced by 50%

*HBV* Hepatitis B virus, *HCC* hepatocellular carcinoma, *DALY* disability-adjusted life years, *ICER* incremental cost-effectiveness ratio, *QALY* quality-adjusted life years, *USD* US dollars, *WHO* World Health Organization

## Financial reasons for expanding treatment indications

Aside from the compelling clinical evidence summarized in the prior section, there are also arguments for treatment indication expansion for financial reasons. While treating more patients would increase medication costs, diagnostic costs may be saved from simplified care pathways, and long-term costs will be saved from reduced HBV-related complications.

### Treating all patients may be cost-effective

Zhang et al. studied 135 scenarios of treatment criteria expansion in China, with all simulated scenarios showing cost-effectiveness by 2050. Among the scenarios, a treat-all strategy with 80% coverage was estimated to save 1,536,561 quality-adjusted life years (QALYs) by 2050 at a cost of 7,160 US dollars per QALY, emerging as the most cost-effective strategy [44]. A similar study simulated a treat-all strategy in the US from 2020 onwards. The treat-all strategy was cost-effective at an annual treatment cost of 2000 US dollars, and can be cost-saving at a treatment cost of 750 US dollars. The interventions could achieve positive economic returns before 2050. Given that annual treatment cost in the US can be as low as 362 US dollars in 2020, a treat-all strategy is financially feasible in the US. [45]

### Cost-effectiveness of other treatment expansion strategies

Aside from the treat-all approach, the cost-effectiveness of other treatment expansion strategies has been studied. Using national data from Korea, Lim et al. studied three strategies of treatment expansion through (1) removing viral load restrictions, (2) lowering ALT cut-off to 1 × ULN, or (3) removing ALT and HBeAg restrictions. By 2035, the 3 strategies averted 11,800, 23,300, and 37,000 liver-related mortalities respectively, and were all highly cost-effective. [46]

A study from Gambia utilized a simplified treatment approach which considered ALT and HBeAg levels only, without requiring HBV viral load requirements. Throughout the lifetime of a modeled cohort of 100,000 CHB patients, the simplified treatment approach averted 4877 disability-adjusted life years (DALYs), and a treat-all approach led to 9352 DALYs averted. In contrast, the EASL 2017 treatment criteria led to an excess of 795 DALYs. The simplified treatment approach was cost-effective at a treatment cost threshold of 352 US dollars, whereas the treat-all approach was not cost-effective. [47]

A 2020 study from Saudi Arabia compared the scenarios of (1) achieving the WHO targets of diagnosing 90% of infections and treating 80% of patients by 2030, and (2) diagnosing and treating all CHB patients by 2022. Achieving WHO targets was associated with 30–35% reduction in HCC and liver-related deaths, whereas a treat-all approach was associated with 50–55% reduction in HCC and liver-related deaths. Achieving WHO targets was estimated to yield a positive economic return, whereas the treat-all approach was only cost-effective if treatment costs could be reduced by 50%. [48]

These cost-effectiveness studies illustrated the potential economic benefits of treatment indication expansion. Nonetheless, local epidemiology and treatment costs would influence whether specific strategies are cost-effective.

## Logistical reasons for expanding treatment indications

The current HBV treatment guidelines are generally catered for specialists, given the need to account for multiple parameters including HBV DNA, ALT, HBeAg, and liver fibrosis [22]. The complexity of current guidelines can be difficult for non-specialists to implement, and the adherence to treatment guidelines is suboptimal in primary care [49, 50]. Furthermore, HBV viral load testing and liver fibrosis assessment may not be routinely available in resource-constrained settings [2]. Expansion and simplification of treatment criteria would remove these treatment barriers to enable wider treatment coverage in different care settings. Positive experiences have been reported from sub-Saharan Africa, where experts applied locally available tests to select candidates for NUC therapy. Through deriving a scoring system with ALT, aspartate aminotransferase (AST) and platelet count only, experts were able to easily select candidates for NUC therapy, enabling decentralization of HBV care to district hospitals. [51]

A treat-all approach may be even easier to implement, as HBsAg detection can be performed by point-of-care testing. This enables counseling and NUC prescription in the index clinic visit, streamlining the process of CHB treatment. Positive experience from a treat-all program has been reported in Uzbekistan [52], and the feasibility of treating all CHB patients has been supported by multiple experts globally. [22, 53, 54]

## Potential caveats of treatment indication expansion

Despite the evidence supporting treatment indication expansion, several caveats must be considered.

### Drug non-compliance

The majority of CHB patients require lifelong therapy after NUC initiation [55, 56], and expanding treatment criteria will lead to a higher cumulative pill-burden in more patients. This may in turn lead to higher incidences of drug non-compliance. Poor compliance to NUCs can lead to virological relapse and biochemical flares, and may theoretically increase the risks of developing antiviral resistance. Furthermore, non-compliance is associated with higher risks of HCC and mortality respectively. [57]

A 2018 meta-analysis pooled data from 23,823 patients, reporting that only 74.6% of CHB patients were adherent to NUC therapy. The medication adherence rates were comparable in different healthcare settings and patient subgroups [58]. Limited knowledge about treatment was a key barrier to drug compliance [58]. As enhanced knowledge on HBV is associated with improved treatment compliance, interventions to enhance patients’ self-efficacy and knowledge will be necessary. [54, 59]

### Safety concerns with long-term NUC therapy

Safety of long-term NUC therapy should also be considered. Current first-line NUCs are generally well-tolerated with favorable safety profiles. However, both TDF and ETV can lead to renal impairment through renal tubular injury [60], with TDF having greater impact on renal function than ETV after long-term treatment [61]. TDF is also associated with reduced bone density and hypophosphatemia after long-term use [60]. The newer option of TAF may have lower bone and renal toxicity, yet TAF may not be as widely available as TDF and ETV. TAF may also confer substantially higher treatment costs, especially in the context of treatment expansion.

### Patient acceptability

Patient acceptability must also be considered before initiation of CHB therapy. Stigmatization toward CHB has been widely reported in different populations, and treatment may lead to further negative labeling [62]. It is hence predictable that some CHB patients prefer to avoid therapy if possible [63, 64]. Despite the common utility of NUCs, research on patients’ attitude toward treatment has remained limited [65, 66]. Better understanding of patients’ perspectives may enable specific strategies to enhance treatment acceptability.

In addition, while NUC therapy can be cost-effective from a public health perspective, some countries still require patients to pay out-of-pocket for NUCs. While generic ETV and TDF are now available at costs as low as 30 USD per year, drug retail costs can vary between nations [45, 67]. Out-of-pocket payment may not be acceptable or affordable for some patients, and this is a known treatment barrier in both high income and low to middle income countries. Global health efforts to subsidize drug costs may be necessary to boost treatment uptake. [68, 69]

## Proposed strategies for expanding treatment indications

**Fig. 2:**
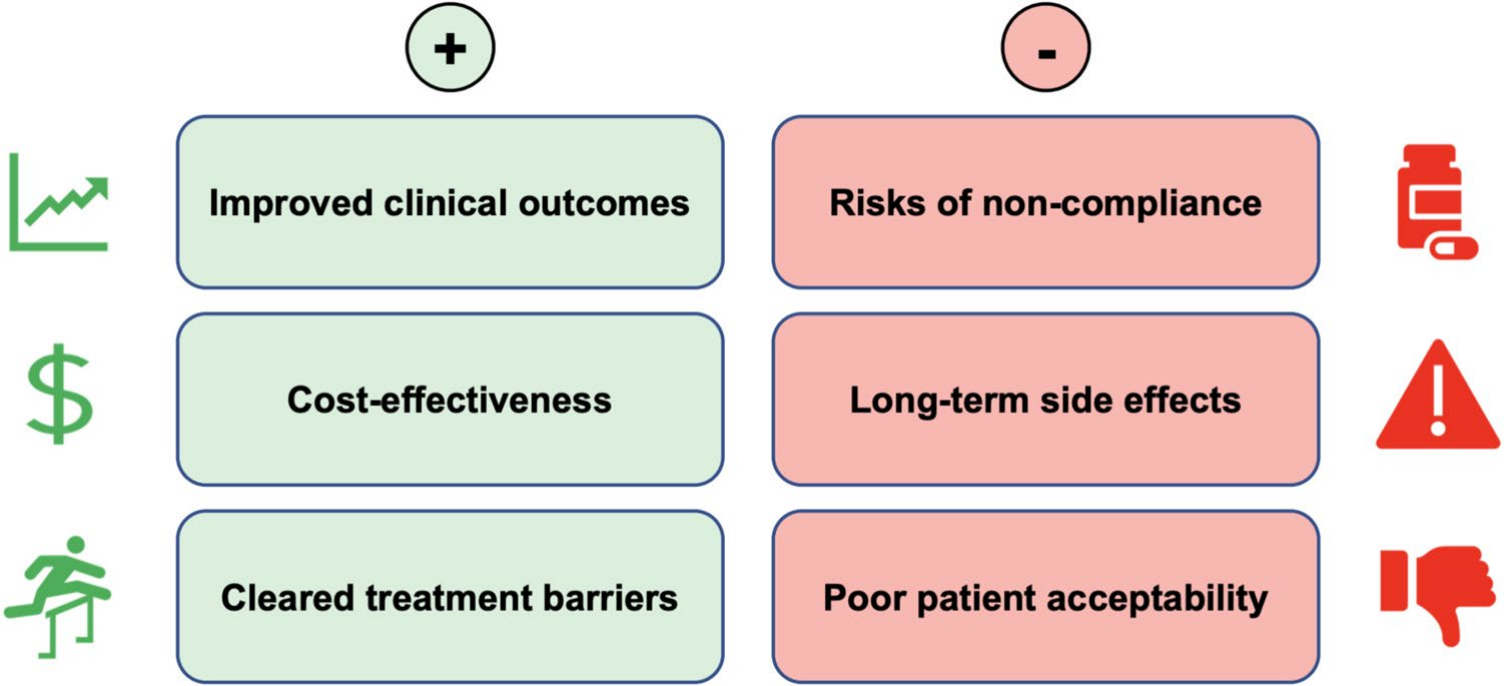
The pros and cons of treatment indication expansion

The pros and cons of treatment indication expansion are summarized in Fig. 2. While the above caveats should not be ignored, they are not insurmountable, and the benefits of treatment expansion outweigh the disadvantages.

There is general consensus among hepatologists in supporting treatment indication expansion, and the WHO 2024 guidelines have already expanded its treatment indications when compared with the AASLD 2018, EASL 2017, and APASL 2016 guidelines respectively. [13–16] Outside of the official guidelines by international societies, multiple expert panels have proposed different strategies for treatment indication expansion. The recommendations by key expert panels are illustrated in Table 3.[17–24] In general, the proposals are straightforward to implement with simple ALT and HBV DNA cut-offs. Of note, the guidelines by the Chinese Society of Hepatology were updated in 2022 [20], and they are one of the societies with the broadest treatment criteria globally. Instead of setting specific HBV DNA cut-offs for treatment initiation, the Chinese guidelines use detectable HBV DNA as a consideration for therapy, which would lead to a considerable increase in treatment-eligible patients.

**Table 3:** Proposed strategies for treatment indication expansion

		Treat-all [17]	Treating viremic or high ALT patients [18]	Treating viremic patients [19]	Chinese Society of Hepatology [20]	North American expert panel [21]	US expert panel [22]	East Asia expert panel [23]	US hepatitis B primary care workgroup^24^
Non-cirrhotic	HBV DNA < 2000 IU/ml + normal ALT	Treat			Treat (in specific scenarios*)	Treat (if significant histologic disease)			
	HBV DNA < 2000 IU/ml + ALT > ULN	Treat	Treat		Treat				
	HBV DNA > 2000 IU/ml + normal ALT	Treat	Treat	Treat	Treat (in specific scenarios*)	Treat (in specific scenarios**)	Treat (if > 30 years old)	Treat (in specific scenarios***)	
	HBV DNA > 2000 IU/ml + ALT > ULN	Treat	Treat	Treat	Treat	Treat	Treat	Treat	Treat
Cirrhosis		Treat	Treat	Treat	Treat	Treat	Treat	Treat	Treat

ALT cut-offs for Chinese Society of Hepatology, North American expert panel, US Expert Panel, and East Asia expert panel: 30 U/L for male, 19 U/L for female

ALT cut-offs for US hepatitis B Primary care workgroup: 35 U/L for male, 25 U/L for female

ALT cut-offs unspecified for the treat-all strategy, treating viremic and high ALT patients strategy, and treating viremic patients strategy

*If family history of HBV-related cirrhosis or HCC, age > 30. Significant inflammation (G ≥ 2) or fibrosis (F ≥ 2), or HBV-related extrahepatic manifestations

**If HBeAg negative with fibrosis. If HBeAg positive, consider HCC risk factors, age, lifestyle, and desire for treatment

***Treat if significant histologic disease. Consider treatment in patients with first-degree family history of cirrhosis or HCC, extrahepatic manifestations, or age > 40 and above

## Discussion—Should we be treating all patients?

Treatment indication expansion is a growing topic of interest in the field of HBV. Patients not indicated for NUC therapy in current guidelines are still at substantial risks of liver-related complications, and expanding treatment to these patients can improve clinical outcomes. Furthermore, treatment indication expansion is cost-effective and can remove treatment barriers. Despite a few caveats which are generally manageable, treatment indication expansion is widely accepted, and will be increasingly adopted globally. When compared with the older guidelines, the WHO 2024 guidelines already represent a large step forward in treatment indication expansion, and should provide guidance for improved clinical practice.

### Treatment expansion strategies must be tailored based on local data

Expert panels have proposed multiple strategies for expanding treatment criteria.

In particular, a treat-all approach has been proposed to yield the greatest benefits from a population health perspective. [44] Since factors including HBV epidemiology, healthcare resource availability, and socioeconomic status vary substantially between countries, a one-size-fits-all approach in unlikely to be feasible. Instead, treatment strategies that are tailored based on local data would be more practical. For example, the simulation studies reported a treat-all approach to be cost-effective in China and in the US [44, 45], but was not cost-effective in Gambia or Saudi Arabia [47, 48], highlighting how regional disparities can influence the feasibility of treatment strategies.

### HBV care pathways and policy implementation must be optimized

Treatment is an important part of HBV care, yet timely diagnosis and linkage to care are equally important toward the goal of hepatitis elimination [70, 71]. As of 2019, only 10.3% of HBV-infected subjects in the world knew of their infection status. In Africa and Southeast Asia, which are highly endemic regions for HBV, the diagnosis rate was as low as 2.2% and 2.1% respectively. [1] Decentralized care models or community health initiatives may be necessary to enhance screening and referral. Aside from screening for HBV, simultaneous screening for HDV may also be necessary in specific populations, as HDV coinfection remains as a challenge in CHB management [72]. Extensive efforts will be required to improve the HBV care pathways to maximize effects from treatment expansion strategies.

It is also important to note that treatment indication expansion may not translate to increased treatment uptake. As of 2022, only 6.8 million out of 83.3 million (8.2%) treatment-eligible patients were treated globally [73]. Healthcare inequalities have been reported in various nations, which in turn become barriers for treatment provision [1, 12]. Rather than solely focusing on strategies of treatment indication expansion, it is critical to ensure that treatment expansion policies can be implemented successfully. Outcome assessment is also essential in ensuring that interventions can translate to tangible benefits for patients. [74]

### Novel antivirals are anticipated to revamp treatment strategies

NUCs are the best treatment options for HBV now, yet HBsAg seroclearance rarely occurs with NUC therapy. Multiple novel antivirals are hence under development with functional cure as the therapeutic goal. The novel antivirals are classified into virus-targeting agents — which inhibit specific pathways in the HBV lifecycle, and immunomodulators — which augment host immunity against HBV [75]. Among these novel antivirals, RNA interference (RNAi) therapeutics are the most advanced in the developmental pathway, and have demonstrated potent and sustainable effects in inducing HBsAg seroclearance [76, 77]. Current research directions have expanded to combination therapies with RNAi therapeutics and immunomodulators, aiming to achieve synergistic effects in HBsAg seroclearance. [78, 79]

Functional cure is associated with improved clinical outcomes [80, 81]. However, HBV integrations persists after HBsAg seroclearance [31], and liver-related complications may still occur [82]. Complete HBV cure, defined as the sterilization of cccDNA and integrated DNA from the human body, is the optimal endpoint in CHB, as it hypothetically eliminates all risks of HBV-related complications [75]. While complete cure is not achievable with current treatment modalities, novel experimental approaches with epigenetic modulation and gene editing have been explored as strategies for achieving complete cure. [83–85] These agents are now entering clinical trials, and the results are keenly anticipated.

Novel antivirals, either targeting functional cure or complete cure, will revolutionize the treatment of CHB. Treatment strategies are anticipated to further evolve once novel antivirals enter routine clinical practice.

### Conclusion

To conclude, accumulating data provides strong rationale for expanding HBV treatment indications. As some of the current evidence is based on modeling data, future studies should aim to provide real-world long-term data regarding treatment indication expansion. Overall, a treat-all approach is viable, yet may not be the optimal strategy in all healthcare settings. Treatment expansion strategies that are tailored based on local data should yield the greatest impact toward hepatitis elimination.

## Data Availability

Data from this manuscript is available from the corresponding author on reasonable request.
